# Quantitative X-ray phase contrast computed tomography with grating interferometry: Biomedical applications of quantitative X-ray grating-based phase contrast computed tomography

**DOI:** 10.1007/s00259-021-05259-6

**Published:** 2021-04-13

**Authors:** Lorenz Birnbacher, Eva-Maria Braig, Daniela Pfeiffer, Franz Pfeiffer, Julia Herzen

**Affiliations:** 1 Physics Department, Munich School of Bioengineering, Technical University of Munich, Munich, Germany; 2 Department of Diagnostic and Interventional Radiology, School of Medicine, Klinikum rechts der Isar, Technical University of Munich, Munich, Germany

**Keywords:** X-ray imaging, Phase contrast imaging, Grating interferometer, Grating interferometry, CT, Computed tomography, Phase contrast tomography, Quantitative imaging, Electron density, Effective atomic number

## Abstract

The ability of biomedical imaging data to be of quantitative nature is getting increasingly important with the ongoing developments in data science. In contrast to conventional attenuation-based X-ray imaging, grating-based phase contrast computed tomography (GBPC-CT) is a phase contrast micro-CT imaging technique that can provide high soft tissue contrast at high spatial resolution. While there is a variety of different phase contrast imaging techniques, GBPC-CT can be applied with laboratory X-ray sources and enables quantitative determination of electron density and effective atomic number. In this review article, we present quantitative GBPC-CT with the focus on biomedical applications.

## Introduction

Phase contrast imaging techniques enable visualization of subtle soft tissue differences in comparison to the conventional attenuation signal. Over the last 25 years, various phase contrast imaging (PCI) methods have been developed and established in modern X-ray imaging enabling high-resolution imaging especially of biomedical samples [1–10]. Different X-ray phase contrast methods include crystal interferometry [11, 12], propagation- based imaging (PBI) [13–17], analyzer-based imaging [18–22], grating interferometry (GI) [23–27], edge illumination (EI) [28–31], or tracking methods [32–35]. Each of those methods presents with individual requirements regarding beam coherence, detector resolution, or need of specific optical elements.

Although PCI originates from highly coherent synchrotron X-ray sources, some of the methods have been successfully translated to laboratory sources with limited coherence like PBI [36], GI [27], EI [29], or tracking methods like speckle-based imaging (SBI) [35]. The combination of phase contrast imaging with computed tomography methods extended the range of phase contrast imaging to three-dimensional sample characterization. This gave rise to numerous biomedical studies on ex vivo tissue samples like brain or lung with PBI [38, 39] next to liver, lung, and esophagus with EI [40], or brain as well as breast with GI [41, 67], to name only a few.

While many imaging applications including phase contrast methods have been focusing mainly on revealing subtle relative signal differences, quantitative imaging is gaining more and more interest. In general, relative contrast signals like the relaxation times in magnetic resonance imaging (MRI) can be sufficient for the desired information. However, absolute signals of physical nature like e.g. the electron density—this is what we describe in this work with quantitativeness—are in theory independent of the method of contrast formation. This does not only increase the comparability and reproducibility of the results but absolute quantitative signals can also be used for standardized diagnostics or for data science on a larger scale [42]. In clinical use for example, quantitative X-ray imaging is emerging shown by the development of dual-energy or spectral computed tomography [43–48]. There, decomposition of spectral CT data into the contributions from photoelectric effect and Compton scattering allows to calculate the electron density, the effective atomic number, virtual monoenergetic images, virtual non-contrast images, iodine maps, or other material decomposition of interest [47]. Clinical applications of spectral CT are for example the quantification of iodine contrast agent for increased tumor discrimination [49–51], enhanced characterization of kidney stones [52, 53], or improved stroke diagnosis [54].

Achieving quantitative phase contrast imaging is however challenging. Among the various PCI techniques available, grating interferometry is not only one of the most sensitive phase contrast methods [55, 56] but the tomographic phase contrast data can also be transferred directly to the electron density. In detail, when performing grating-based phase contrast computed tomography (GBPC-CT), one can convert the experimentally determined phase contrast signal to the electron density. In the case of polychromatic, laboratory sources’ additional effective energy calibration has to be applied [57–59]. This allows the determination of an absolute physical quantity, which enables comparing electron density results of different GI setups with different imaging methods like dual-energy CT or with reference and theoretical values [60]. Application of quantitative GBPC-CT covers myocardial infarct [61], atherosclerotic plaque [62], and tumors of kidney [63], liver [64], pancreas [65], brain [67–69], testis [70], and breast [41, 57, 72, 73]. Moreover, biochemical or biomedical fluids can be characterized quantitatively [66, 74]. In addition, Hounsfield units for the phase (HUp) [57, 75] or the effective atomic number *Z*_eff_ can be calculated [76–78]. The complementarity of the attenuation and phase signal of GBPC-CT can be used to perform material decomposition [79] or a decomposition similar to spectral CT into electron density and effective atomic number [80–82].

In this review, we will focus on quantitative imaging with grating-based phase contrast tomography. In comparison to other phase contrast imaging techniques, GBPC-CT enables quantitative high spatial resolution imaging not only at synchrotron sources, but also relatively robust at polychromatic laboratory high-flux X-ray sources. After a presentation of the theoretical basics of X-ray phase contrast imaging, we will outline how we can determine electron density and effective atomic number with monochromatic and even polychromatic X-ray sources using GBPC-CT. This results in various biomedical applications of GPBC-CT and a brief presentation of related X-ray PCI methods in context of ability to provide quantitative data.

## Phase contrast imaging

The quantity underlying changes in the phase *Φ* of electromagnetic waves is the complex index of refraction *n*. The different phase contrast methods can either directly access phase differences *Φ* of electromagnetic waves like in crystal interferometry, the gradient of the phase ∇*Φ* like in ABI, EI, GI, and tracking-based methods, or the second derivative of the phase ∇^2^*Φ* as measured in PBI.

### Complex index of refraction

**Fig. 1:**
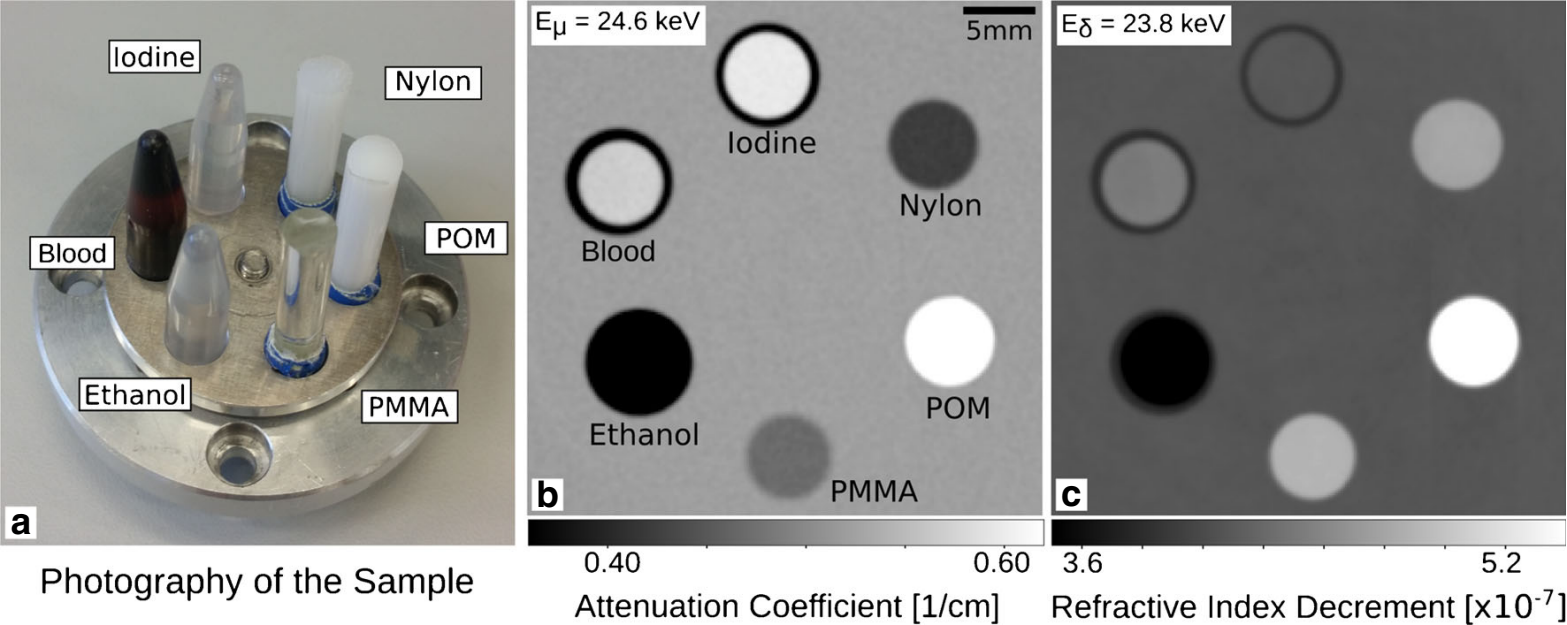
Quantitative characterization and decomposition of a phantom measured with GBPC-CT at the Munich Compact Light Source (MuCLS) [80]. The materials are depicted in subfigure (**a**). Tomographic attenuation (**b**) and phase contrast data (**c**) can provide complementary information as further presented in Fig. 8 and Table 1. The phase contrast signal is proportional to the electron density. Figure adapted from [80]. This figure is licensed under the Creative Commons Attribution (CC BY)

The complex index of refraction is a material specific quantity comprised by the refractive index decrement *δ* and the imaginary part of the refractive index *β*

1 $$ n(E) = 1 - \delta(E) + i\beta (E), $$

and depends on the energy of the electromagnetic wave *E* [4, 84]. Figure 1 illustrates the complementarity of the attenuation coefficient *μ* and the refractive index decrement *δ* of an exemplary GBPC-CT phantom measurement. The imaginary part of the refractive index *β* is related to the attenuation coefficient *μ* as

2 $$ \beta = \frac{\mu}{2k}, $$

with *k* = 2*π*/*λ* being the magnitude of the wave number. The refractive index decrement *δ* represents the phase-shift properties and is related to the electron density *ρ*_e_ in absence of absorption edges as

3 $$ \delta = \frac{2\pi r_{0} \hbar^{2} c^{2} }{E^{2}} \rho_{\mathrm{e}},  $$

where *r*_0_ is the classical electron radius, $\hbar $ is the reduced Planck constant, and *c* is the speed of light. In theory, the electron density can be calculated if the mass density *ρ* and composition of the material of interest are known [57]:

4 $$ \rho_{\mathrm{e}} = \rho N_{\mathrm{A}}\frac{\sum w_{i} Z_{i}}{\sum w_{i} A_{i}}.  $$

The weights *w*_*i*_ account for the fraction of atom *i*. *Z*_*i*_ represents the atomic number, *A*_*i*_ the atomic mass number, and *N*_A_ Avogadro’s number. Changes in the phase induced by an object in the X-ray beam are proportional to the refractive index decrement *δ* as:

5 $$ {\varPhi} = \int k \cdot \delta \mathrm{d}z  $$

## Grating interferometry

**Fig. 2:**
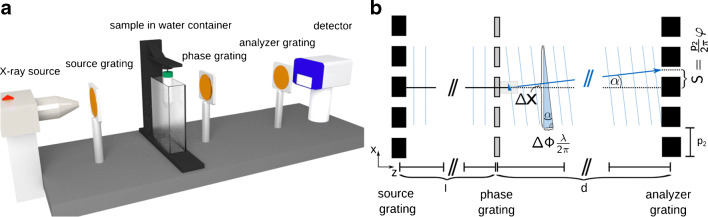
Schematic laboratory GBPC-CT setup (**a**) and illustration of the physical signal forming process of a three grating interferometer (**b**). The setup consists of the X-ray source and three X-ray gratings, namely the source grating, the phase grating, and the analyzer grating, followed by an X-ray detector. The sample is put into a water container to reduce the so-called phase-wrapping. In the schematic on the right, refraction in the sample causes a change of the direction of the wave front *α*, which is proportional to the difference in optical path *ΔΦ*/(2*π*) ⋅ *λ* over a lateral beam distance *Δx*. The changed wave front propagates to the analyzer grating causing a shift in the interference pattern *S*. The figure on the left is licensed under the Creative Commons Attribution (CC BY) and first published in Willner et al. [74]

The focus of this review lies on quantitative grating-based phase contrast computed tomography (GBPC-CT). A laboratory GBPC-CT setup is illustrated in Fig. 2a. In detail, an X-ray Talbot interferometer uses the Talbot self-imaging effect, which has first been described for reappearing periodic structures with visible light and originates from Fresnel diffraction [83–85]. At specific distances *d* from those periodic structures, e.g., an optical grating, a moiré interference pattern appears. The period of the pattern *p* depends on the optical grating type which can in the case of X-rays be categorized as an attenuation or a phase grating, usually inducing a phase shift of either *Δπ* or *Δπ*/2. Due to angular refraction, a sample in the beam creates a lateral shift *S* of the interference pattern. For X-rays, the angular shift is typically in the order of micro-radians. Therefore, another grating—the so-called analyzer grating—is used to resolve the lateral shift with common detector pixel sizes and reasonable propagation distances. By moving one of the gratings in discrete steps over one period of the interference pattern, the lateral shift induced by a sample can be extracted as the lateral phase-shift of the resulting stepping curve *φ*. This process is called phase stepping [25].

This way, three different signals can be retrieved with GI, the conventional attenuation signal *T*, the differential phase contrast *φ*, and the dark-field signal, which represents the coherence reduction by the sample [23, 24, 86, 87]. More on the dark-field small-angle scattering signal can be found in Pfeiffer et al. [87]. There are also other extraction methods of the phase shift like Fourier transform–based single shot methods using one- or two-dimensional gratings [88, 89].

The phase shift *φ* of the interference pattern is related to the phase shift *Φ* of the wave front via the refraction angle *α* using small-angle approximation:

6 $$ \frac{1}{k}\frac{{\varDelta} {\varPhi}}{{\varDelta} x} = \frac{p_{2}}{2\pi d} \varphi, $$

with *p*_2_ being the period of the analyzer grating and *d* the distance between the phase and the analyzer grating. An illustration of the effects taking place is shown in Fig. 2b. The phase shift *Φ* can then be directly related to the refractive index decrement *δ* as already shown in Eq. 5.

### Signal extraction and CT reconstruction

#### Phase stepping

Phase stepping is recommended for stable retrieval of the differential phase contrast (DPC) signal *φ* for high angular sensitivity and thus high electron density resolution. The measured intensity within the phase-stepping approach is a convolution of the source shape, the intensity pattern, and the attenuation profile of the analyzer grating. This intensity in dependency of the grating position *x*_g_ can be approximated to

7 $$  I(x,y,x_{\mathrm{g}}) \approx a_{0} + a_{1} \sin \left( \frac{2\pi }{p}x_{\mathrm{g}} + \varphi \right). $$

Two phase-stepping curves are measured—one without the sample being the reference scan and one with the sample [25].

#### Attenuation contrast

The attenuation contrast is the average value of the intensity *a*_0_ of the stepping curve. Including sample and reference scan, the transmission signal is

8 $$  T(x,y) = \frac{a_{0}^{\mathrm{s}}}{a_{0}^{\mathrm{r}}} = \mathrm{e}^{-\int \mu(x,y,z)\mathrm{d}z}, $$

with superscript “s” denoting the sample scan and superscript “r” the reference scan.

#### Differential phase contrast

The two stepping curves for the sample and the reference have different phase offsets. The difference between these signals is the differential phase contrast (DPC) signal

9 $$  \varphi(x,y) = \varphi^{\mathrm{s}} - \varphi^{\mathrm{r}}. $$

Advanced signal extraction methods to reduce jitter manifested in the stepping curve can be applied for homogeneous artifact-free DPC projections [90–94]. Additional phase ramp correction of the raw DPC signal is often needed to retrieve homogeneous DPC projections [95]. In order to reduce phase-wrapping artifacts, which occur if the phase shift is larger than 2*π*, putting the sample in a water container reduces phase contrast differences at the sample borders and thus also minimizes phase-wrapping artifacts [58, 59]. In addition, spectral phase unwrapping algorithms can be applied [96, 97].

#### Computed tomography

Grating interferometry can be combined with computed tomography [25, 26, 67]. The differential phase contrast projections can be reconstructed using filtered backprojection (FBP) with a Hilbert filter for phase integration [37]. Noise analysis in GBPC-CT reconstructions using FBP has been performed intensively in Raupach and Flohr [98], Köhler et al. [99], Chen et al. [100], and Weber et al. [101]. In direct combination with computed tomography, one can also apply reverse projection methods [67, 102–104] or phase contrast tomography with interlaced phase steps [105]. Moreover, statistical iterative reconstruction methods can be used for grating-based phase contrast computed tomography (GBPC-CT) when noise has to be reduced or the number of projections is limited [106–109] or the reconstruction should be performed without phase stepping [110, 111]. One can furthermore increase GBPC-CT image quality with bilateral filtering of the phase and the attenuation signal [112].

### Laboratory GBPC-CT

GI can be used in a laboratory environment by implementing an additional grating directly behind the X-ray source as illustrated in Fig. 2a. This so-called source grating is an absorption grating which provides sufficient partial coherence for the method to work with extended X-ray sources like clinical X-ray tubes [27]. As an alternative to the source grating, one can also utilize micro focus X-ray source with small focal spot sizes [113, 114] or a structured anode [115, 116].

However, when using polychromatic X-rays, the performance of GBPC-CT suffers from spectral dependencies and from beam hardening as known from conventional attenuation CT [117–119]. The already presented water container acts also as beam filtration reducing spectral differences between the measurements with and without sample. Eventually one measures the relative differential phase contrast (DPC) signal. According to the relations shown in Eqs. 3 and 5, the relative DPC signal can be converted into the relative refractive index decrement *δ*_rel_. In order to get absolute quantitative values, the corresponding refractive index decrement of the water has to be added to the experimentally determined relative signal *δ*_rel_ as

10 $$ \delta_{\text{abs}} = \delta_{\text{rel}} + \delta_{\mathrm{H_{2}O}} (E_{\text{eff}}).  $$

The value of $\delta _{\mathrm {H_{2}O}} (E_{\text {eff}})$ is determined based on the electron density of water of 334e/n*m*^3^ and the effective energy *E*_eff_ [58, 59], see Eq. 3. Although the determination of quantitative values is requires a monochromatic spectrum as available at monochromatic synchrotron sources, the determination of accurate quantitative values is also possible with polychromatic sources [57–59, 76]. Therefore, an effective interaction energy has to be determined, either by calibration materials or by defining a mean energy from measured or simulated spectra. While the energy of the usually monochromatic beam at a synchrotron is known, one has to determine an effective energy for polychromatic X-ray sources. One way to determine the effective energy in the latter case is to use calibration materials like PMMA [57]. As a final GBPC-CT result, the electron density is calculated based on Eq. 3

11 $$ \rho_{\mathrm{e}} = \frac{E_{\text{eff}}^{2} }{2\pi r_{0} \hbar^{2} c^{2} } \delta_{\text{abs}}.  $$

### Hounsfield units

In clinical CT, relative quantification of the attenuation is performed using the so-called Hounsfield units (HU), which express the attenuation compared to water

12 $$ HU = \frac{\mu_{\text{sample}} - \mu_{\mathrm{H_{2}O}} }{\mu_{\mathrm{H_{2}O}} - \mu_{\text{air}} } \times 1000,  $$

with the energy-dependent attenuation coefficients of water and air. Although the HU values are still energy dependent, this definition allows the comparison of HU values from measurements at different clinical CT systems in a range that is sufficient for reliable diagnostics. In a similar manner, Hounsfield units for the phase contrast signal can be defined based on the refractive index decrement *δ* [74, 75]:

13 $$ \begin{array}{@{}rcl@{}} HU_{\mathrm{p}} =\frac{\delta_{\text{sample}} - \delta_{\mathrm{H_{2}O}} }{\delta_{\mathrm{H_{2}O}} - \delta_{\text{air}} } \times 1000 . \end{array} $$

The definition of HUp provides a normalized physical quantity comparable to the well known clinical HU. In contrast to HU, HUp do not show energy dependence and are therefore truly comparable between different setups.

### Effective atomic number

With the electron density and attenuation coefficient at hand, as it is the case with GBPC-CT, one can determine also the effective atomic number *Z*_eff_ as shown in refs. [57, 76, 78, 81]. The effective atomic number is like the electron density an absolute physical quantity that can be used for material characterization and decomposition, although theoretical calculation of the effective atomic number varies [120–122].

For calculation of the effective atomic number *Z*_eff_, there are two main approaches. In Qi et al. [76], the experimentally determined refractive index and attenuation coefficient are used for a calibration-based exponential fit of the effective atomic number:

14 $$ \begin{array}{@{}rcl@{}} \frac{\mu }{\rho_{\mathrm{e}}} = p \cdot Z_{\text{eff}}^{n} + q, \end{array} $$

with the three fit parameters *p*, *q*, and *n*. The fit parameters have to be determined in a prior GBPC-CT calibration experiment of a phantom with the same spectrum as the later GBPC-CT measurement of the sample and known reliable (effective) atomic number. The effective atomic number is then accessed by comparing the experimental ratio on the left side with the fit data.

In the method presented by Willner et al. [57], one can avoid this additional calibration step by using tabulated cross section data. The attenuation coefficient *μ* is related to the total atomic interaction cross section as

15 $$ \begin{array}{@{}rcl@{}} \mu = \rho \frac{N_{\mathrm{A}}}{A} \sigma_{\text{tot}} (E,Z), \end{array} $$

where *ρ* is the mass density and *N*_A_ is Avogadro’s constant. The total atomic interaction cross section *σ*_tot_(*E*,*Z*) depends on both the energy and the atomic number and is proportional to the electronic cross section as *σ*_tot_(*E*,*Z*) = *Z* ⋅ *σ*_e,tot_(*E*,*Z*). Rearranging 15 in combination with the formula for the electron density shown in Eq. 4 leads to

16 $$ \begin{array}{@{}rcl@{}} \frac{\rho_{\mathrm{e}} }{\mu } = \frac{Z_{\text{eff}}}{\sigma_{\text{tot}} (E,Z) }. \end{array} $$

The ratio on the left side is determined in a GBPC-CT experiment and compared to tabulated data on the right side [123–127], which is interpolated to access effective atomic numbers *Z*_eff_.

### High angular sensitivity in GBPC-CT

Reaching high electron density resolution with GBPC-CT is challenging, especially in the laboratory case. Depending on the desired application and spatial resolution, the performance of a GBPC-CT setup depends on numerous factors. Next to the choice of X-ray source and detector, the design of the GBPC-CT setup with position of gratings and sample have to be optimized [56, 119, 128–133]. The gratings form central elements of the interferometer featuring periods in the micrometer range. While phase gratings are comparably easy to fabricate, absorption gratings for the relevant X-ray energies are challenging to fabricate as they need high attenuating lamellae at small periods [23, 134–138]. Photon counting detectors [139–141] and spectral detectors [142] are able to reduce noise in GBPC-CT and thus increase the electron density resolution [82, 143].

Experimentally, the electron density resolution *Δρ*_e_ can be determined based on the standard deviation of the reconstructed refractive index decrement in a homogeneous sample volume as

17 $$ {\varDelta} \rho_{\mathrm{e}} = \frac{E_{\text{eff}}^{2} }{2\pi r_{0} \hbar^{2} c^{2} } \sigma_{\delta_{\text{abs}}},  $$

similar to Eq. 11.

## Applications of quantitative GBPC-CT

**Fig. 3:**
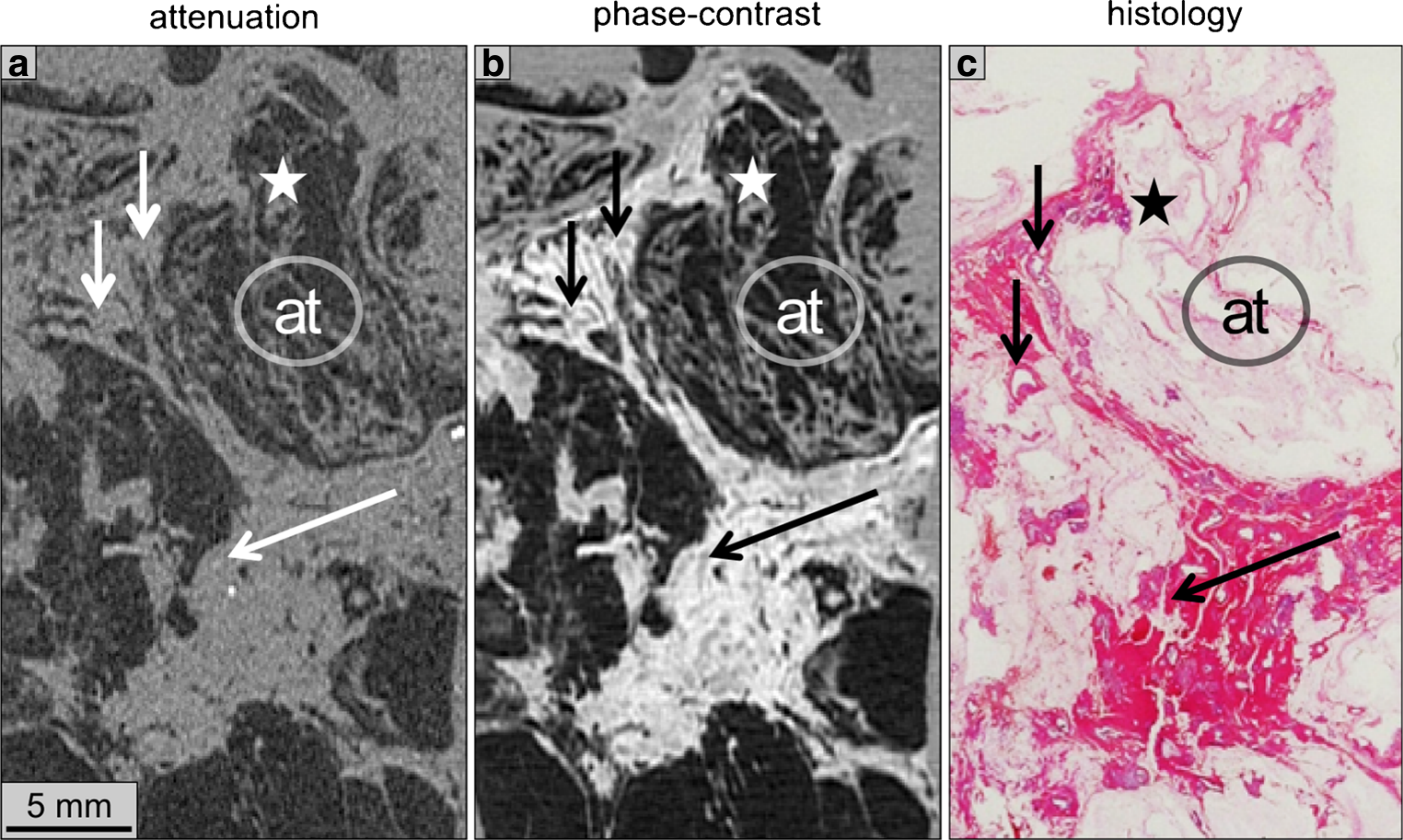
Exemplary illustration of the differences of attenuation contrast (**a**) and phase contrast (**b**) of a human breast cancer sample in comparison to histopathology (**c**). The soft tissue detail in the phase contrast slice (**b**) is much higher than in the corresponding attenuation image (**a**). The patient was suffering from ductal carcinoma in situ (DCIS) as can be seen by the dilated ducts in the phase contrast image marked with arrows. The ex vivo sample was measured with laboratory grating-based phase contrast computed tomography (GBPC-CT). Further details can be found in the original article by Hellerhoff et al. [41]. This figure is licensed under the Creative Commons Attribution (CC BY)

Due to the increased soft tissue contrast at high resolution, GBPC-CT is a promising technique for biomedical imaging. Various biomedical studies have shown applications of quantitative GBPC micro-CT of ex vivo samples at both synchrotron and laboratory ranging from the quantitative classification of different soft tissues and body fluids to the visualization of pathological changes in biopsy samples. While specific applications also aim towards clinical in vivo imaging, most studies presented in this section focus on small ex vivo tissue samples, which could be used for improving clinical histology or to address basic research questions (Fig. 3).

One of the first quantitative GBPC-CT imaging results are shown in Pfeiffer et al. [67], which features a rat brain with an induced gliosarcoma tumor. The presented electron density resolution of this synchrotron measurement was 0.18e/n*m*^3^, while the contrast in the corresponding attenuation signal was limited. Several further GBPC-CT experiments from synchrotron sources have illustrated the potential of quantitative biomedical imaging [105, 144–149]. It was even possible to visualize human cerebellar structures with high quantitative soft tissue contrast comparable to MRI but at much higher spatial resolution [68, 69]. High-resolution GBPC-CT imaging at subtle soft tissue differentiation based on the electron density allowed a big step towards X-ray 3D quantitative phase contrast histology as published in Zanette et al. [70]. More recent results by Thalmann et al. allow to differentiate quantitatively nerve fibers at high spatial resolution [71].

The transition of quantitative GBPC-CT results from synchrotron facilities to laboratory X-ray sources allowed an impact in research activity extending the range of applications [58, 75, 132]. In Herzen et al., first characterization of fluids was performed in a laboratory environment where the quantitative material values of the GBPC-CT were validated with tabulated reference values. By that the complementarity of phase and attenuation and the feasibility of quantitative measurements at a conventional X-ray source were demonstrated [59]. It could also be shown that GBPC-CT at a polychromatic source operating at 70 kVp, which is comparably high, provides good quantitative results [150]. However, increasing the performance of GI for higher energies depends strongly on grating fabrication. Absorption gratings at small periods and large attenuation material thickness of the lamellae are technically challenging [138]. Laboratory GI setups operating at 160 kVp have been successfully realized as proof of concept [151].

**Fig. 4:**
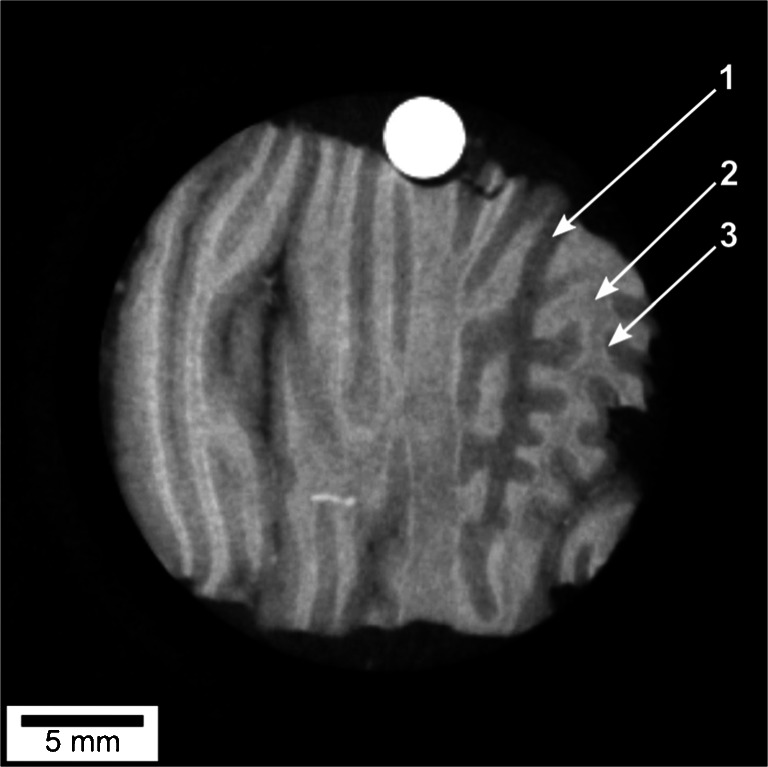
Tomographic GBPC-CT slice of a human cerebellum sample. The stratum moleculare (1) can be differentiated from the stratum granulosum (3) and white matter (2) [56]. This figure is licensed under the Creative Commons Attribution (CC BY)

Further development of GBPC-CT imaging increased the performance with respect to the electron density resolution in the lab [56, 141, 152, 153]. In Birnbacher et al., highly sensitive electron density resolution could be realized with laboratory sources, as visualized by the discrimination of cerebellar tissue in Fig. 4. The electron density resolution was 0.45e/n*m*^3^, which is comparable to synchrotron experiments [56].

**Fig. 5:**
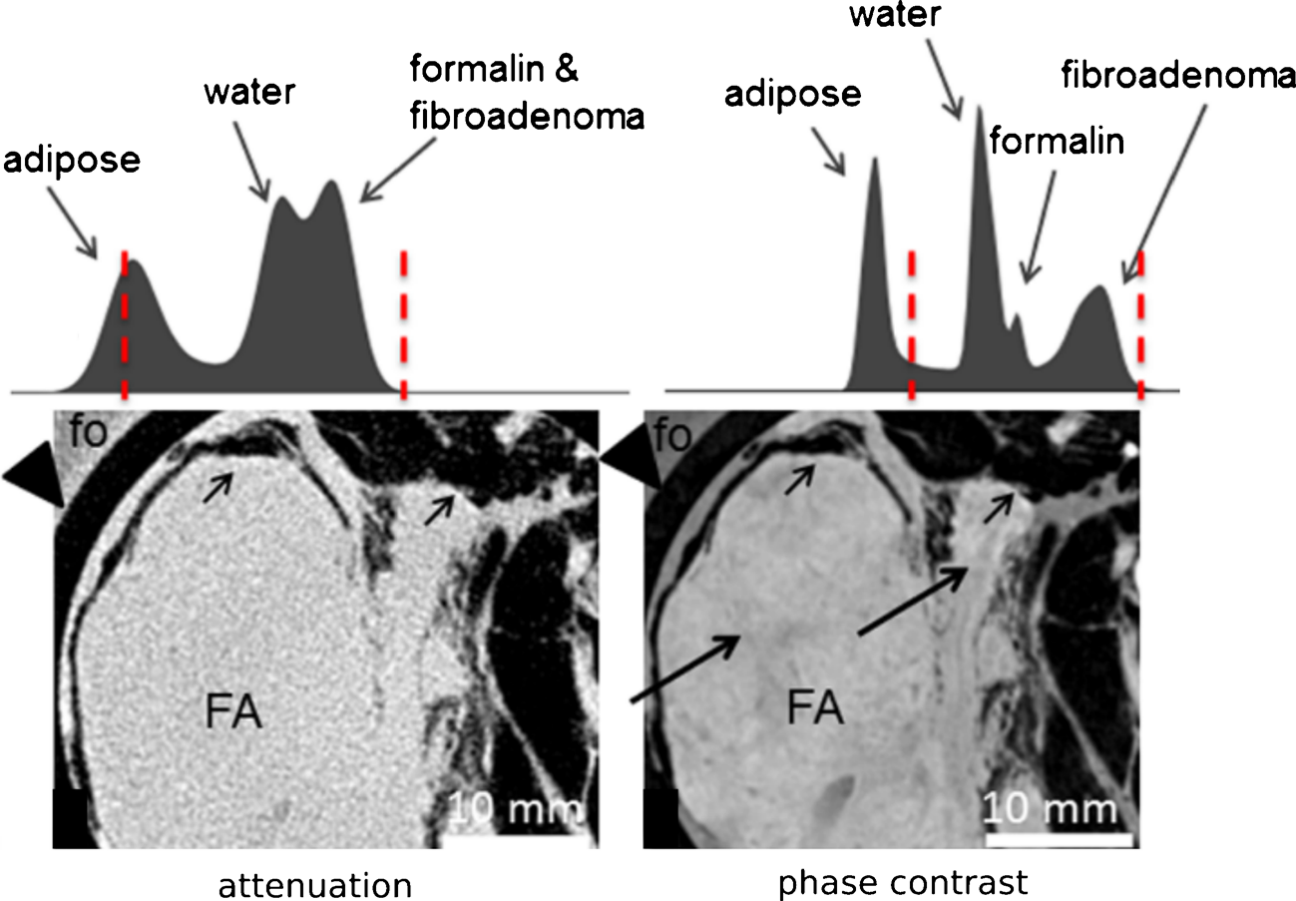
Quantitative GBPC-CT characterization of a fibroadenoma breast sample published in Grandl et al. [73]. The attenuation (left) and phase contrast (right) signal in sagittal view are depicted. Mammary ducts are indicated by long arrows, while short arrows mark adhering adipose tissue. The corresponding histograms show the distribution of the HU and HUp values of the respective contrast signal of the complete volume. The distinction in the phase contrast signal is much higher than in the corresponding attenuation signal. The window levels are indicated by the dashed red lines. Figure adapted from [73]. This figure is licensed under the Creative Commons Attribution (CC BY)

One focus of high-resolution quantitative GBPC-CT imaging is breast imaging. Early architectural changes of breast cancer or its precursor forms can be visualized based on the electron density with both synchrotron and laboratory studies [41, 72]. Willner et al. demonstrated the clear differentiation of adipose and fibroglandular tissue based on their HUp, where a differentiation in conventional CT would not be possible due to very similar HU [57]. A further study investigated the discrimination of fibroadenoma from other breast lesions also based on HUp values [73] (see Fig. 5). The corresponding histograms illustrate the clear separation of tissues of interest. Exemplary quantitative GBPC-CT data of a ductal carcinoma in situ (DCIS) sample is illustrated in Fig. 3. In comparison to the corresponding attenuation image, which does not reveal specific soft tissue contrast like thickening of the ductal wall, the phase contrast image reveals similar findings as the corresponding histological image [41].

**Fig. 6:**
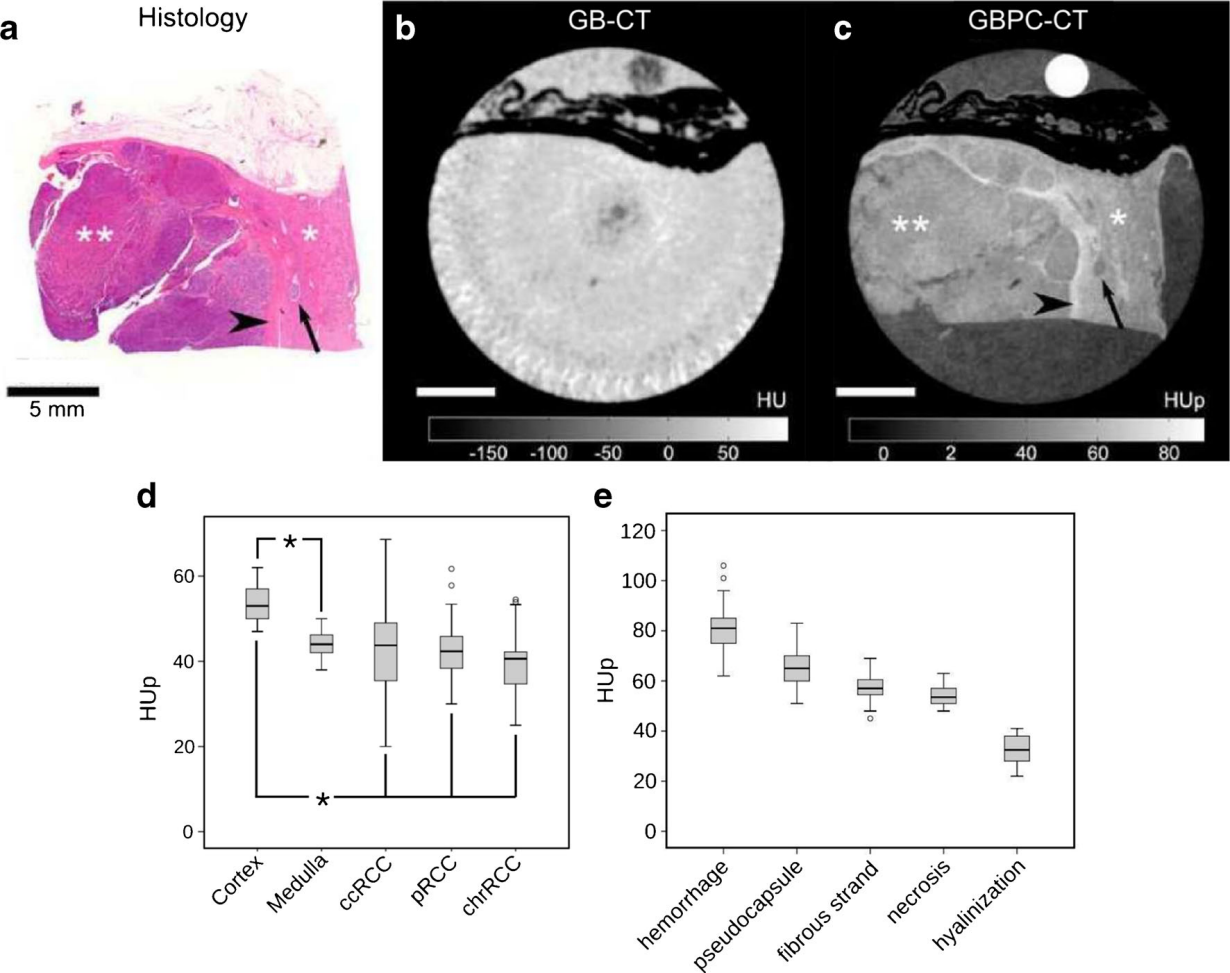
A papillary renal cell carcinoma (RCC) sample imaged with a GBPC-CT setup is shown by way of example. The corresponding histological slice (**a**) is presented next to the tomographic slice of the attenuation contrast (**b**) and phase contrast (**c**) signal. A clear discrimination between healthy (*) and tumorous (**) renal cortex is visualized in the phase contrast signal, which is not the case for the attenuation signal. The arrowhead points to a pseudo-capsule around the tumor which was also not revealed in the attenuation signal. In subfigure (**d**), the HUp values of different RCC types (ccRCC: clear cell, pRCC: papillary, chrRCC chromophobe RCC) as well as cortex and medulla are shown. Subfigure (**e**) depicts quantitatively different RCC features. Figure adapted from Braunagel et al. [63]. This figure is licensed under the Creative Commons Attribution (CC BY)

Atherosclerotic plaque was analyzed quantitatively in Hetterich et al. [62]. There, calcified, lipid-rich, and fibrous tissue associated with the formation of atherosclerotic plaque was characterized. Similar results were published in Winklhofer et al. [154] and in Bonnano et al. [155]. In Notohamiprodjo et al., HUp changes in myocardial tissue types were compared with grades of myocardial lesions [61]. A multi-modal comparison of different imaging methods for the characterization of cartilage degeneration revealed additional quantitative information on morphology and ultra-structure by GBPC-CT compared to MRI [156]. Khimchenko et al. presented electron density GBPC-CT images of cartilage and bone using an adapted commercial micro-CT system [114]. Perfusion of ischemic kidneys was analyzed with X-ray phase contrast imaging in [157]. In Braunagel et al., renal cell carcinoma components were analyzed with GBPC-CT to characterize the subtypes clear cell, papillary, and chromophobe renal carcinoma based on HUp values [63]. Exemplary GBPC-CT slices compared with histology and quantitative HUp results of this study are depicted in Fig. 6.

With the development of compact inverse Compton sources, the gap between large scale synchrotron facilities and laboratory sources has been closed. These sources provide quasi-monochromatic X-rays with tunable energy [158]. Eggl et al. [159] demonstrated tomographic GBPC-CT experiments at an early prototype of such a compact light source, which allowed the visualization of brown adipose tissue in mice [160]. Similar results were achieved at a laboratory setup, which demonstrated transition from brown to white adipose tissue in mice utilizing three-dimensional anatomical electron density distribution. The biochemical changes in adipose tissue could thereby be related quantitatively to the changes in mitochondrial concentration [161].

**Fig. 7:**
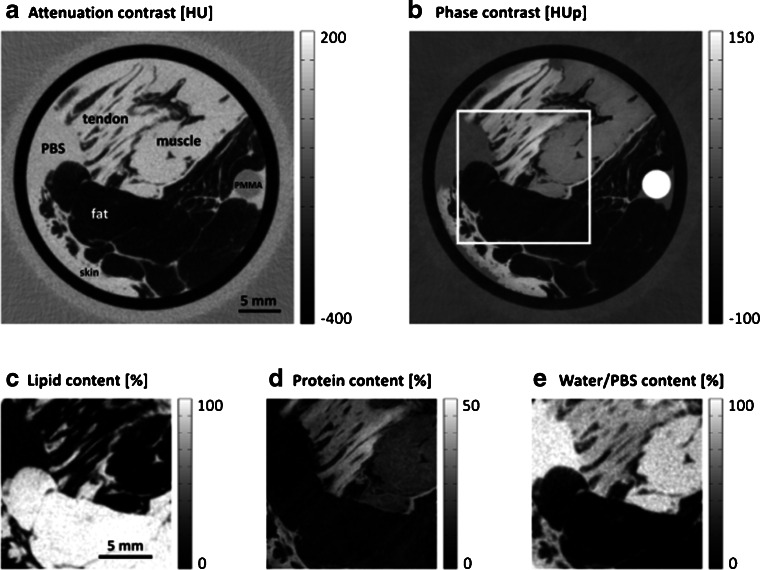
GBPC-CT slices of attenuation (**a**) and phase contrast (**b**) of soft tissue for decomposition into lipid, protein, and water. The axial tomographic slices of the attenuation (**a**) and phase contrast (**b**) signal contain tendon, muscle, fat, and skin tissues. The decomposition into lipid (**c**), protein (**d**), and water (**e**) is displayed for the region marked by the white box in (**b**) [79]. This figure is licensed under the Creative Commons Attribution (CC BY)

Since GBPC-CT setups also enable the determination of the attenuation coefficient, this additional information can be used for quantitative material decomposition based on signal complementarity. In Willner et al. [79], a decomposition of GBPC-CT data based on phase contrast and attenuation data into lipid, protein, and water allowed to quantify the components of soft tissue, which is visualized by way of example in Fig. 7. Quantitative mass density measurements of differences in human teeth composition including dentin and enamel were realized in synchrotron experiments at 53 keV. The mass density was thereby approximated based on the electron density [162]. An analysis study of blood and pathological fluids complemented research in that direction. Different types of infectious fluids were differentiated based on their composition like cell, protein, and water content [66]. GBPC-CT experiments are typically performed with fixed samples to prevent degradation. The tissue fixation with formaldehyde or phosphate buffered saline (PBS) changes quantitative values slightly in comparison to reference electron density values [60, 74]. A similar GBPC-CT study of simulated liver lesion analyzed fluids based on the renal cyst composition (protein rich, hemorrhagic, and enhanced) [64].

**Fig. 8:**
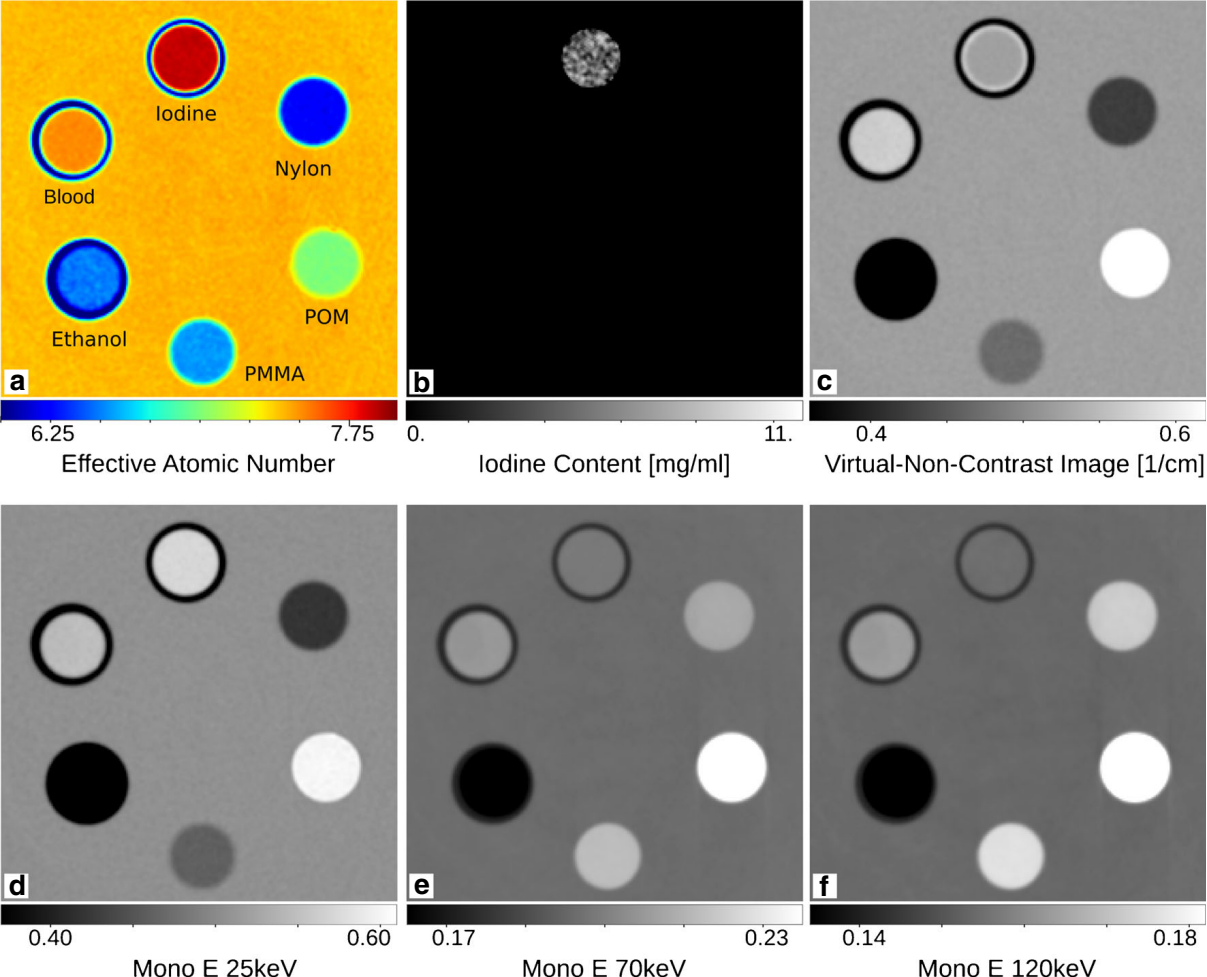
Visualization of the effective atomic number map (**a**), the iodine map (**b**), the virtual unenhanced image (**c**), and virtual monoenergetic images for three different energies (**d**–**f**) using GBPC-CT. The materials are depicted in Fig. 1 next to the attenuation coefficient and refractive index, which is proportional to the electron density. The representation of this data is based on dual-energy CT. Figure adapted from Braig et al. [80]. This figure is licensed under the Creative Commons Attribution (CC BY)

As already mentioned, the combination of the electron density and the attenuation coefficient enables calculation of the effective atomic number [57, 76, 78]. As the measured attenuation and phase contrast signals deliver two independent data sets, they can be used for quantitative material decomposition as known from dual-energy imaging. The calculation of iodine maps, virtual non-contrast images, and virtual monoenergetic images has been demonstrated with measurements at the Munich Compact Light Source (MuCLS) [80], as illustrated in Figs. 1 and 8. Corresponding GBPC-CT quantitative values of the phantom used in this work are presented in Table 1.

**Table 1:** Quantitative values obtained in GBPC-CT as presented in Figs. 1 and 8

Material	*μ*_m_[c*m*^− 1^]	*μ*_l_[c*m*^− 1^]	*δ*_m_[10^− 7^]	*δ*_l_[10^− 7^]	*Z*_eff,m_	$Z_{\mathrm {eff, l_{1}}}$	$Z_{\mathrm {eff, l_{2}}}$	*ρ*_e,m_[10^29^*m*^− 3^]	*ρ*_e,l_[10^29^*m*^− 3^]
NaI	0.597 ± 0.006	0.609	4.08 ± 0.07	4.07	7.94 ± 0.06	–	–	3.34 ± 0.07	–
Blood	0.568 ± 0.006	0.574	4.31 ± 0.07	4.27	7.60 ± 0.06	–	7.74	3.54 ± 0.08	–
Ethanol	0.323 ± 0.006	0.325	3.23 ± 0.06	3.27	6.52 ± 0.07	6.35	–	2.69 ± 0.07	2.68
PMMA	0.470 ± 0.006	0.470	4.70 ± 0.06	4.70	6.58 ± 0.03	6.47	6.56	3.86 ± 0.06	3.86
POM	0.628 ± 0.006	0.628	5.55 ± 0.06	5.55	7.05 ± 0.05	6.95	7.03	4.56 ± 0.06	4.56
Nylon	0.423 ± 0.006	0.419	4.61 ± 0.06	4.58	6.24 ± 0.06	6.12	6.21	3.79 ± 0.06	3.76
Water	0.523 ± 0.006	0.523	4.07 ± 0.06	4.07	7.51 ± 0.05	7.42	7.51	3.34 ± 0.06	3.34

The table shows quantitative results of the attenuation coefficient *μ* and refractive index decrement *δ* as well as the determined electron density *ρ*_e_ and effective atomic number *Z*_eff_ compared to reference data marked by “l.” Experimental GBPC-CT data is marked by “m.” Data adapted from Braig et al. (CC BY) [80]

In Braig et al., three-material decomposition was performed using electron density, effective atomic number, and the dark-field signal provided by laboratory GBPC-CT. The study presented there lead to precise calculation iodine contrast agent concentration [81]. A further step is the combination of spectral detector information with GBPC-CT, which enables noise reduction and consequent increase in quantitative electron density resolution [143]. Latest results of laboratory GBPC-CT combined with a spectral detector with two energy bins improved contrast enhancement in three-material GBPC-CT decomposition [82].

## Related phase contrast imaging methods

In this section, we present shortly related PCI techniques and their ability to provide quantitative results since GBPC-CT is only one among many X-ray PCI methods.

Free-space propagation-based imaging (PBI) [13–17] can be achieved either with multiple distances as so-called holotomography [163, 164] or as a single distance experiment [165]. High resolution close to histology can be achieved with PBI [166]. However, with single distance PBI tomography, the quantitativeness of heterogeneous samples is limited [55]. PBI does not only work with highly coherent synchrotron sources but also with polychromatic laboratory sources like micro focus [36, 167, 168] or liquid jet sources [38, 39]. Generally speaking for PBI, the source size and the detector pixel size should be relatively small; hence, clinical X-ray imaging components cannot be used. With respect to quantitative imaging, the broad spectrum does not limit the application, but cone beam geometry renders quantitative multi-distance phase contrast results more challenging. One large research focus is breast imaging including tomography [169, 170].

In analyzer-based imaging (ABI) or diffraction enhanced imaging (DEI), the X-ray beam is refracted by a crystal, which is rotated for maximum intensity forming a so-called rocking curve. A sample in the beam changes this curve, which is related to the differential phase contrast signal of the sample [18–22]. Application of ABI includes especially many studies of breast imaging in projection and tomography [171–174]. To our knowledge, ABI is available only at synchrotron facilities and does not provide absolute quantitative values.

Edge illumination (EI) or coded aperture is a non- interferometric phase contrast method. Two gratings serve as sample and detector mask and allow the determination of the refractive angle and thus the differential phase signal [28–31]. The method features relaxed requirements in comparison to GI with respect to the grating periods and can be applied with X-ray sources with large focal spots, which renders the method interesting for the use of clinical X-ray sources [28, 29]. Quantitative EI can be realized either by two full individual scans in opposite direction or within a certain range of accuracy in a single scan if the sample attenuation is low [175]. Applications include breast imaging [176] or tomography of biological samples [177]. Quantitative EI tomography also works with laboratory X-ray sources [40].

Speckle-based X-ray phase contrast imaging uses a wave front modulator pattern, which is changed by an object in the beam. This modulator can be quite simple like e.g. sandpaper [35]. Compared to this, the fabrication of the gratings in grating interferometry is quite challenging [178]. This tracking-based method works with polychromatic sources; however, microfocus sources have to be used [35, 178–180]. Promising tomographic electron density results of biomedical samples were shown at high spatial resolution, however only at synchrotron sources [181–183]. More information can be found in the review articles presented in the introduction of this article.

## Conclusion

X-ray grating-based phase contrast computed tomography provides the energy-independent quantities electron density and effective atomic number. The use of a source grating enables the extraction of the material quantities with high accuracy at laboratory sources. GBPC-CT data can also be converted into phase contrast Hounsfield units (HUp) in similarity to the conventional energy-dependent Hounsfield units HU in clinical CT. This possibility for improved tissue characterization based on absolute quantitative values increases the range of phase contrast micro-CT aside from mere relative contrast-based images. In this review, we summarized theory and applications of quantitative GBPC-CT imaging.

The range of GBPC-CT method goes from differentiation of pathological tissue like myocardial infarct or atherosclerotic plaque to tumors of kidney, brain, and especially breast. Thereby, subtle differences in electron density can be determined. Moreover, biochemical or biomedical fluids can be characterized quantitatively. The complementarity between the attenuation and phase signal improves the distinction of objects and enables quantitative material decomposition. Additional consideration of the dark-field signal allows the decomposition into up to three materials.

A potential application in this direction could be pre-histology GBPC-CT, where quantitative GBPC-CT data could be used for advanced tissue characterization leading to more precise and efficient sectioning in histology. Having the ability to access absolute quantitative GBPC-CT data could be used for deep learning and could also lead to improved histological diagnosis [184–186].

While many studies focus on tissue characterization in GBPC micro-CT at high spatial and electron density resolution, one promising development of GBPC-CT towards clinical application could be phase contrast breast tomography [187, 188]. The needed energy range of breast imaging is still in a range feasible for complementarity of the attenuation and phase contrast signal, as the tube voltage for breast phase contrast CT would be lower than conventional CT tube voltages. In combination with spectral detectors and iterative reconstruction, this would allow quantitative assessment of breast tissue and characterization of breast lesions [143].

In order for GBPC-CT to play a larger role in high-resolution biomedical micro-CT imaging, the spatial resolution laboratory environment has to increase aside from further optimization with respect to imaging time. Yet, the availability of the electron density with high accuracy renders GBPC-CT eminently interesting for quantitative biomedical and material science micro-CT applications. Further development of spectral detectors [143], using a combination of analyzer grating and detector [189], or the implementation of a dual phase grating approach [190–192] is expected to increase the performance of laboratory GBPC-CT in the near future.
